# Dinosaur trackways from the Upper Cretaceous Nichkesai Formation near Mayluu Suu City, Southern Tien Shan Mountains, north-western Kyrgyzstan

**DOI:** 10.1098/rsos.230311

**Published:** 2023-05-24

**Authors:** Joseph T. Flannery-Sutherland, Ilja Kogan, Yaroslav S. Trubin, Peter L. Falkingham, Alina Winkler, David Donner De Sousa, Kiril D. Krylov, Anastasia A. Pokhaznikova, Meerim Derbisheva, Tamas Kapitany, Alexey Dudashvili

**Affiliations:** ^1^ School of Earth Sciences, University of Bristol, Bristol, UK; ^2^ Museum für Naturkunde Chemnitz, Chemnitz, Germany; ^3^ TU Bergakademie Freiberg, Freiberg, Sachsen, Germany; ^4^ Laboratory of Sedimentology and Paleobiosphere Evolution, University of Tyumen, Russia; ^5^ Institut für Geowissenschaften, Universität Bonn, Bonn, Germany; ^6^ School of Biological and Environmental Sciences, Liverpool John Moores University, Liverpool, UK; ^7^ Department of Applied Geology and Environmental Sciences, American University of Central Asia, Biskhek, Kyrgyzstan; ^8^ National Dinosaur Museum, Canberra, Australia; ^9^ Tian-Shan Geological Society, Bishkek, Kyrgyzstan

**Keywords:** Campanian, Maastrichtian, Kyrgyzstan, dinosaur, tidal flat, photogrammetry

## Abstract

Trackways provide essential data on the biogeographic distribution, locomotion and behaviour of dinosaurs. Cretaceous dinosaur trackways are abundant in the Americas, Europe, North Africa and East Asia, but are less well documented in Central Asia despite extensive exposure of Cretaceous terrestrial sedimentary rocks in the region. Here we report the presence of bipedal, tridactyl dinosaur trackways near the city of Mayluu Suu, Jalal Abad Oblast, north-western Kyrgyzstan, the first discovery of dinosaur trace fossils within the country. The trackways are situated on a steep slope uncovered by a landslide around the year 2000 in a highly landslide-affected area. Photogrammetry is used to digitally analyse and conserve the trace fossils. We infer a shoreface setting for the trackways based on the locality sedimentology, discuss the identity of the track makers and highlight the potential for future trackway discovery in the area. This discovery contributes vital data to an otherwise sparse record on the spatio-temporal distribution of dinosaurs in Kyrgyzstan, and to the dinosaur trackway record of Central Asia.

## 1. Introduction

Dinosaurs were abundant, diverse and globally distributed during the Cretaceous. Their body and trace fossil records document remarkable morphological and ecological disparity in response to ecosystem transformation during the Cretaceous Terrestrial Revolution [1]. These ecosystems are well documented in North and South America, Europe, North Africa and East Asia, but Central Asian dinosaur faunas are poorly sampled and understudied by comparison [2]. Body fossil material indicates that Central Asian dinosaur assemblages in the Cretaceous contained a diverse mixture of theropods, sauropods and ornithopods [3,4] and biogeographic analyses highlight their importance to dinosaur macroevolution [5,6]. In particular, the origin of tyrannosaurids in Central and East Asia [7,8], followed by their dispersal into North America, was responsible for the remarkable community structure of dinosaur-dominated ecosystems in these regions [9,10].

The frequently fragmentary nature and relative paucity of dinosaur body fossils from Central Asia hinders greater understanding of terrestrial ecosystems during the Jurassic and Cretaceous, despite extensive exposure of terrestrial Mesozoic sediments in the region by the late Cenozoic uplift of the Tien Shan mountain range [11]. In addition, discoveries of many dinosaur-bearing localities were recorded in specialist Soviet literature which is often unavailable to the wider scientific community and compounded by the problems of transliteration and naming conventions between Cyrillic and Roman alphabets (e.g. Киргизстан, Kirghizia, Kirghizstan, Kyrgyzstan) [12,13]. To date, stegosaurians, tetanuran theropods, an ornithischian with possible pachycephalosaurid affinity, and the large-bodied sauropod *Ferganosaurus verzilini* from the Callovian Balabansai Formation [14,15]; eggs from the Aptian-Albian Khodzhaosman Formation, Santonian Yalovach Formation and Campanian-Maastrichtian Nichkesai Formation [16]; an ornithomimid from the Albian-Cenomanian Tokubai Formation [17]; and instances of indeterminate hadrosaur, sauropod and theropod bones from other Jurassic-Cretaceous-aged localities (approx. 50) [2,12,18] provide widespread, if often fragmentary, evidence for diverse dinosaurian faunas in Kyrgyzstan.

**Figure 1.**
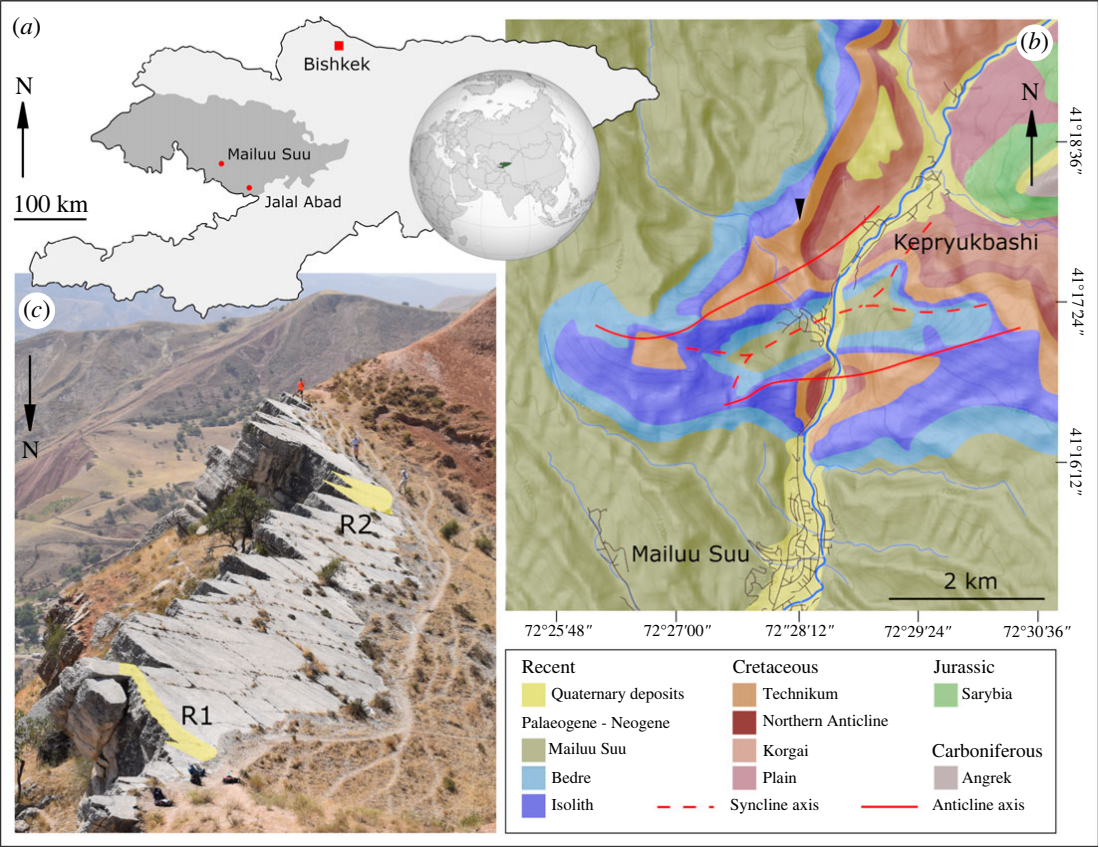
Geographical and stratigraphic setting of the dinosaur trackways. (*a*) Location of Mailuu Suu city, Jalal Abad oblast within southwestern Kyrgyzstan. (*b*) Geological map of the key tectonic structures and formations exposed in the locality area, redrawn from Schlogel *et al*. [31] (based on de Marneffe [32]). The black triangle marks the location of the track site. (*c*) South-facing photograph of the entire track site, highlighting the location of the two-principal track-bearing areas. Three rucksacks are present in the foreground of this photograph and the track surface is approximately 100 m long.

Despite its potential for yielding new discoveries of dinosaurian material, poor sampling strongly affects the trace fossil record of Central Asian dinosaurs [19] compared to well-documented, abundant trackway assemblages in other parts of the globe. Trackways are currently known from Uzbekistan, Turkmenistan, Tajikistan and the Tien Shan mountains in Qinghai-Tibet, China, most of which are Jurassic in age (table 1). We add to this record with the discovery of sedimentary structures from the Upper Cretaceous of southwestern Kyrgyzstan (figure 1) which we interpret here as dinosaur trackways, the first described from the country and a vital addition to the fossil record of Kyrgyz dinosaurs.

**Table 1.** Summary of Central Asian dinosaur track sites.

site	location	age	source
Hojapil-Ata	Turkmenistan	Oxfordian	Amnniyazov [20]
Ak-Gaya	Turkmenistan	Oxfordian	Fanti *et al*. [21]
Chagyl	Turkmenistan	Bajocian	Elfimov *et al*. [22]
Bolshoi Balkhan	Turkmenistan	Upper Jurassic	Amnniyazov *et al*. [23]
Khodzha - Karshavar	Turkmenistan	Upper Jurassic	Amnniyazov *et al*. [23]
Tashkurgan I	Uzbekistan	Oxfordian	Gabunyia & Kurbatov [24]
Tashkurgan II	Uzbekistan	Oxfordian	Meyer & Lockley [19]
Tangiduval I	Uzbekistan	Oxfordian	Meyer & Lockley [19]
Tangiduval II	Uzbekistan	Oxfordian	Meyer & Lockley [19]
Tangiduval III	Uzbekistan	Oxfordian	Meyer & Lockley [19]
Derbent I	Uzbekistan	Hauterivian	Meyer & Lockley [19]
Derbent II	Uzbekistan	Hauterivian	Meyer & Lockley [19]
Ravat	Tajikistan	Middle Jurassic	Rozdestvensky [25]
Shirkent I	Tajikistan	Cenomanian	Zhakarov [26]
Shirkent II	Tajikistan	Oxfordian	Gabunyia & Kurbatov [27]
Kharkush	Tajikistan	Tithonian	Dzhalilov & Novikov [28]
Khingou	Tajikistan	Upper Jurassic	Dzhalilov & Novikov [28]
Babatag I	Tajikistan	Upper Albian	Amnniyazov *et al*. [23]
Babatag II	Tajikistan	Upper Albian	Bulin *et al*. [29]
Yangguang	Qinghai - Tibet	Hauterivian - Berriasian	Xing *et al*. [30]
Mailuu Suu	Kyrgyzstan	Upper Cretaceous	this study

## 2. Geological setting

The Mayluu Suu area exposes largely Cretaceous and Cenozoic sedimentary rocks deposited across the northeastern margin of the Fergana basin, with biostratigraphic age control primarily derived from bivalves entering the basin during eastward marine incursions of the proto-Paratethys [33–36]. The trace-bearing locality (figure 1) was discovered in 2001 by Alexey Dudashvilli (last author) and Apas Bakirov following its exposure by a landslide. The surface is 100 m long and 15 m wide at its greatest extent and sits at the crest of an anticlinal ridge located 2 km north of Mayluu Suu city (41°18′10″ N, 72°28′18″ E, 1348 m elevation), dipping 30° and striking 300° west.

By older stratigraphic literature, the locality exposes the Campanian-Maastrichtian Nichkesai Formation [34]. As this formation name is used elsewhere in reference to dinosaur fossils from the Jalal Abad region [2,12,18], it is our preferred stratigraphic designation. More recent mapping efforts established an alternate set of unit names in the area [31,32]. While these units do not follow formal stratigraphic naming conventions, their ages at the locality section are compatible with the stratigraphic range for the Nichkesai Formation and their designation has utility for assessment of landslide risk in the area which may affect future fossil discovery (see discussion). By this newer mapping effort, the trace-bearing surface occurs in the top sandy limestones of the Upper Cretaceous Technikum ‘formation’ [31,32]. This unit is covered by clayey sandstone with thin limestone beds of the Palaeocene-aged Isolith and underlapped by limestone of the Upper Cretaceous Northern Anticline ‘formations’ [31,32]. The western slope of the ridge is covered by landslide deposits, while the sheer eastern side exposes the anticline structure, with uranium mine tailings and the remains of the Mayluu Suu enrichment plant at its base in the valley floor. Several large blocks of the trace-bearing surface have fallen eastwards since its exposure.

## 3. Material and methods

### 3.1. Sedimentological documentation

The locality surface was examined to identify all areas with sedimentary structures. The fallen blocks were additionally investigated for structures, but none were found either due to genuine absence or inaccessibility of any trace-bearing surfaces. The sedimentary succession containing the trace-bearing surface was logged to permit inference of its depositional setting. A further section approximately 150 m north of the trace-bearing surface and around 20 m above the trackway surface in the stratigraphic succession was also investigated due to its sedimentary relevance.

### 3.2. Photogrammetric analysis

The trace-bearing surface was examined in detail to identify all structures present. Selected sets of traces were documented using photogrammetry to permit digital analysis of the structures and for conservation purposes given the high likelihood of trace loss through weathering or slumping of vulnerable blocks. Photographs were taken in bright sunlight following the procedural recommendations of Matthews *et al*. [37] using a Canon EOS 6D camera with a Canon EF 24-105 mm lens, a focal length of 50 mm and ISO 200. 418 and 601 photographs were taken of two key trace-bearing areas (figure 1*c*). Photogrammetric reconstructions were generated from each set of images using Meshroom (v. 2021.1.0) with default settings. The models were cleaned and manually aligned with the horizontal plane in Blender (v. 3.1.2) and Meshlab (v. 2022.02) then false colour relief maps generated from the cleaned models using CloudCompare (v. 2.13). Where photogrammetric reconstructions and photographs were used to produce interpretative drawings of the traces, all three sets of images are presented in line with the principles of best practice described by Falkingham *et al*. [38]. All photographs and digital models are archived on Zenodo [39]. Finally, linear measurements of each set of traces (table 2) were made from the false colour relief maps using ImageJ (v. 1.53).

**Table 2.** Linear measurements of each set of tracks (cm).

track and side	pedal length	pace length	left stride	right stride
*R1 – T1*				
1, R	32	81	—	162
2, L	41	85	164	—
3, R	36	85	—	157
4, L	41	78	156	—
5, R	43	84	—	163
6, L	43	78	160	—
7, R	34	86	—	171
8, L	32	86	—	—
9, R	37	—	—	—
*R2 – T1*				
1, L	14	68	129	—
2, R	17	65	—	125
3, L	15	63	125	—
4, R	16	67	—	125
5, L	12	64	130	—
6, R	15	71	—	131
7, L	15	65	128	—
8, R	14	68	—	128
9, L	20	66	122	—
10, R	18	62	—	127
11, L	14	72	130	—
12, R	16	61	—	123
13, L	21	66	126	—
14, R	21	62	—	123
15, L	16	66	121	—
16, R	19	63	—	—
17, L	14	—	—	—
*R2 – T2*				
1, L	14	43	93	—
2, R	22	58	—	113
3, L	15	60	95	—
4, R	19	47	—	45
5, LR	—	110	106	108
6, LR	—	61	71	59
7, R	27	23	—	84
8, L	20	68	115	—
9, R	17	58	—	99
10, L	20	49	80	—
11, R	20	37	—	—
12, L	16	—	—	—
*R2 – T3*				
1, R	15	66	—	128
2, L	18	65	131	—
3, R	17	68	—	129
4, L	16	63	—	—
5, R	17	—	—	—
*R2 – T4*				
1, R	16	62	—	116
2, L	17	61	117	—
3, R	19	62	—	122
4, L	18	63	—	—
5, R	15	—	—	—

## 4. Results

### 4.1. Locality sedimentology

**Figure 2.**
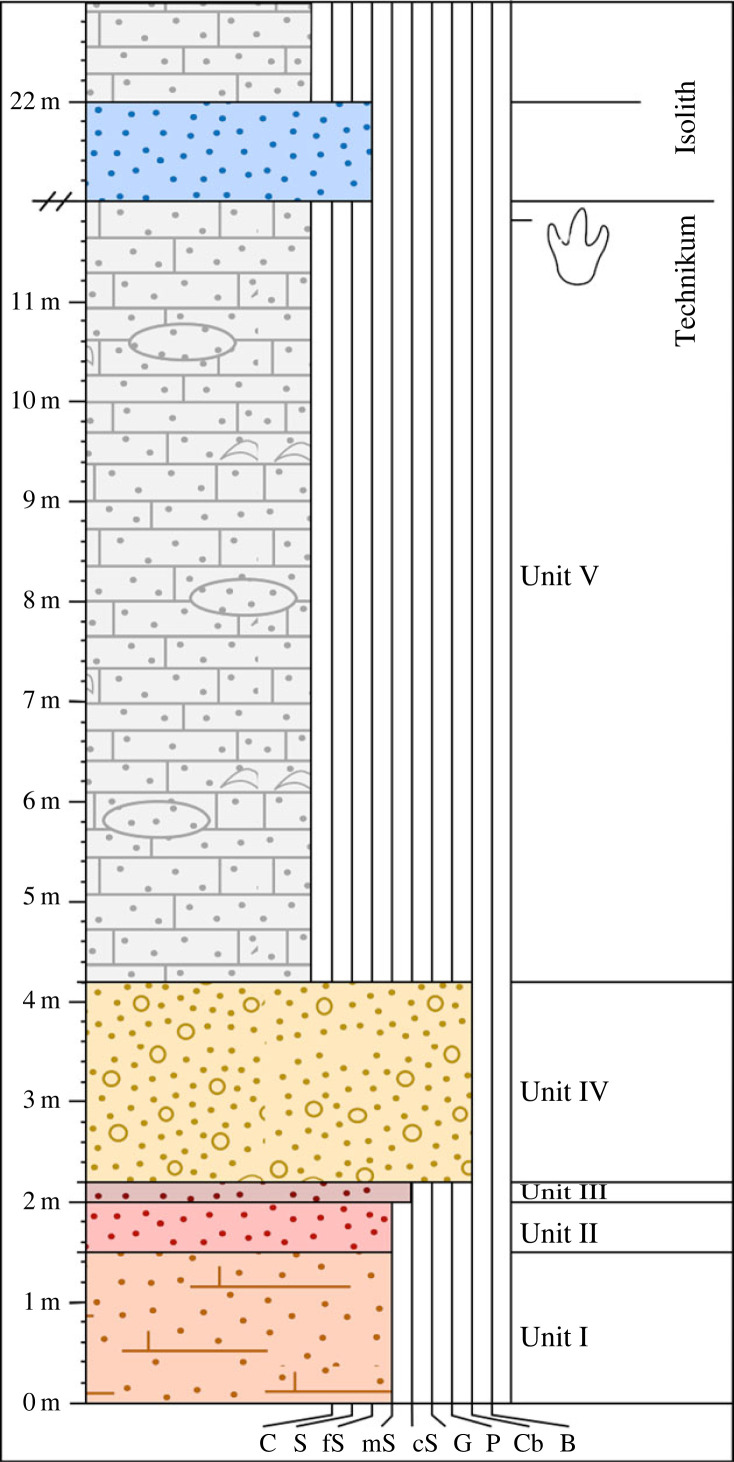
Sedimentary succession at the dinosaur trackway site. The sedimentological log records the transition between the Upper Cretaceous Technikum Formation and the Palaeogene Isolith Formation and highlights the level of the track-bearing surface. The lowermost 20 m of the Isolith Formation are omitted at the schematic discontinuity beginning at 12 m up the section, but this is composed of same sandstone lithology recorded up to 22 m above the base of the logs. C, clay; S, silt; fS, fine sand; mS, medium sand; cS, coarse sand; G, gravel; P, pebble; Cb, cobble; B, boulder.

A 12-metre sedimentary log of the stratigraphic succession at the locality was made (figure 2). All described units share the strike and dip of the trace-bearing surface and show conformable contacts.

Unit I (150 cm): blocky, jointed, well cemented, grey to light brown silty sandstones with calcrete nodules and thin coarse sandstone lenses containing subangular to subrounded gravel. The unit represents the uppermost portion of a thicker succession of the same facies comprising the bulk of the anticline.

Unit II (46 cm): well cemented purple and grey silty sandstone with concentric iron stains and rust spots.

Unit III (20 cm): coarse dark brown silty sandstone with subrounded to rounded clasts. Clasts coarsen upwards from gravel at the base of the unit to cobbles at the top.

Unit IV (200 cm): matrix-supported, polymict conglomerate composed of angular to rounded cobbles and infrequent bivalved shells. The matrix is composed of coarse sandstone with gravel.

Unit V (760 cm): thickly bedded, jointed grey limestone. Shell-rich beds are present at the base of the unit (20 cm) and halfway through (10 cm). Subangular to subrounded clastic grains are distributed throughout the unit, concentrated into sandy lenses within the limestone in some places. The trace-bearing surface is near the very top of this unit and is capped by no more than 30 cm of rubbly, highly weathered limestone.

The trace-bearing surface is overlain by red, yellow and pale grey silty sandstones Approximately 20 m of this facies is present until another limestone bed is reached. Cherty gravel becomes increasingly prevalent in the top 80 cm of the sandstone facies and transitions smoothly into grey limestone (100 cm) comparable to Unit V. No sedimentary structures were identified on the surface of this bed.

### 4.2. Trace morphology

**Figure 3.**
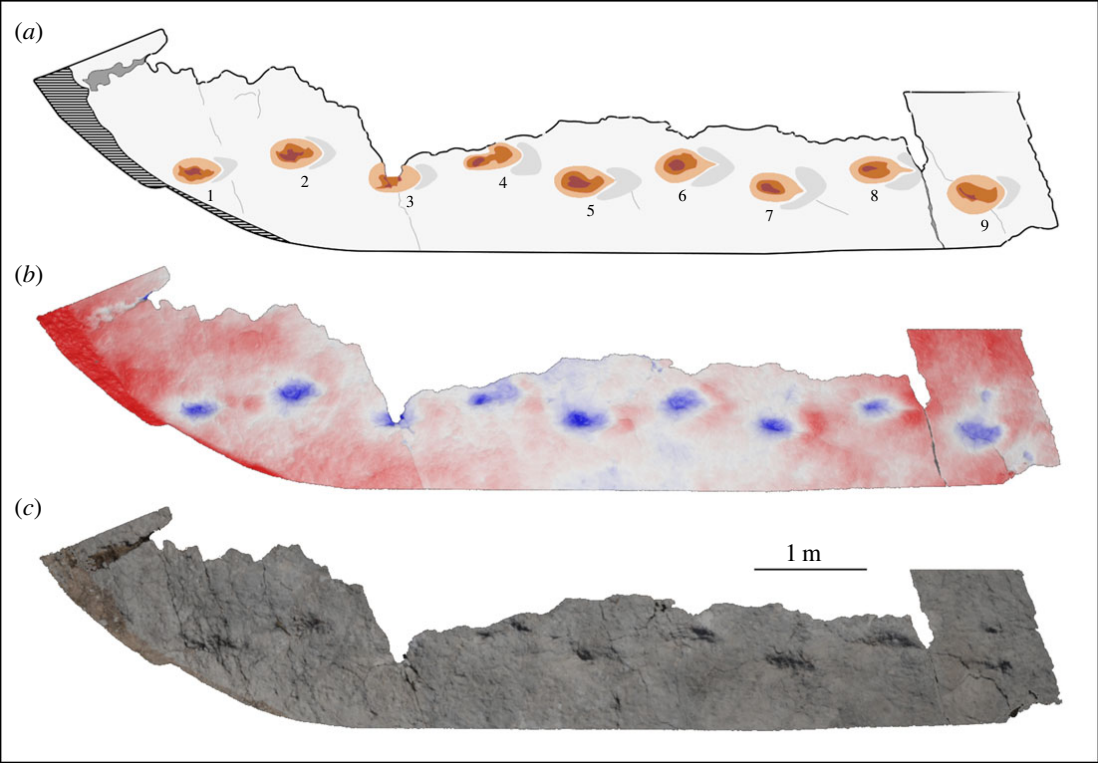
R1 dinosaur trackway surface at Mailuu Suu. Ascending track numbers indicate the directionality of the trackway. The hatched area demarcates vegetated rubbly soil obscuring the foot of the bedding place. (*a*) Schematic interpretation of a false colour relief map of the R1 surface made with photogrammetry. (*b*) False colour relief map. (*c*) True colour photogrammetric reconstruction.

**Figure 4.**
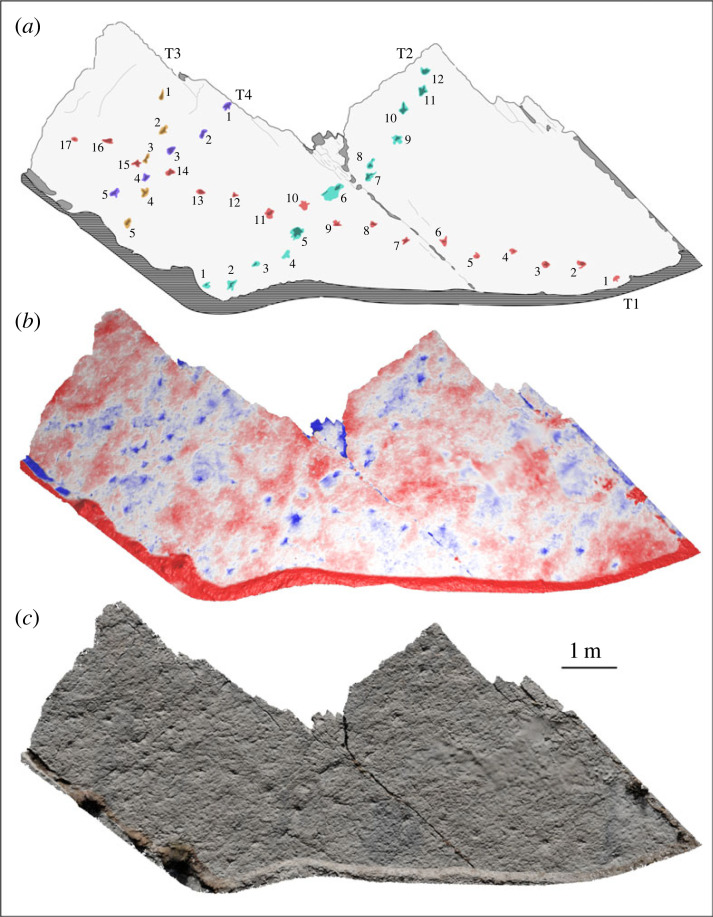
R2 dinosaur trackway surface at Mailuu Suu. Ascending track numbers indicate the directionalities of each trackway. The teal, red, yellow and purple tracks correspond to the T1, T2, T3 and T4 trackways, respectively. The hatched area demarcates vegetated rubbly soil obscuring the foot of the bedding place. (*a*) Schematic interpretation of a false colour relief map of the R1 surface made with photogrammetry. (*b*) False colour relief map. (*c*) True colour photogrammetric reconstruction.

The trace surface bears jointing cracks (figure 1*c*) but is relatively smooth and displays no sedimentary structures other than the traces documented here. Two principal trace-bearing regions of the surface were identified, designated here as R1 and R2 (figure 1*c*), alongside scattered, isolated examples across the rest of the surface. The traces lack anatomical fidelity because of formational processes in soft substrate [40] with margins which merge smoothly into the bedding surface. In the principal trace-bearing regions, the traces are arranged linearly with the long axis of the ovate depressions aligned with this lineation, and an alternating offset of each trace to either side of the lineation axis (figures 3 and 4). Some traces display remnants of potentially more complex morphologies, leading us to characterize them here as tracks and trackways based on their regularity (table 3) and the lack of confounding sedimentary structures on the bedding plane.

**Table 3.** Track measurements (mean, standard deviation) and calculated biomechanics.

trackway	pedal length (cm)	stride (cm)	hip height (cm)	body length (cm)	gait
R1 – 1	37.6 (s.d. = 4.5)	161.9 (s.d. = 5.0)	187.7	378.9	0.86
R2 – 1	16.2 (s.d. = 2.6)	126.6 (s.d. = 3.1)	73.2	292.8	1.73
R2 – 2	19.0 (s.d. = 3.8)	90.1 (s.d. = 21.1)	87.8	351.2	1.02
R2 – 3	16.6 (s.d. = 1.1)	129.3 (s.d. = 1.5	75.2	303.2	1.72
R2 – 4	17.0 (s.d. = 1.6)	118.3 (s.d. = 3.2)	77.3	309.2	1.53

**Figure 5.**
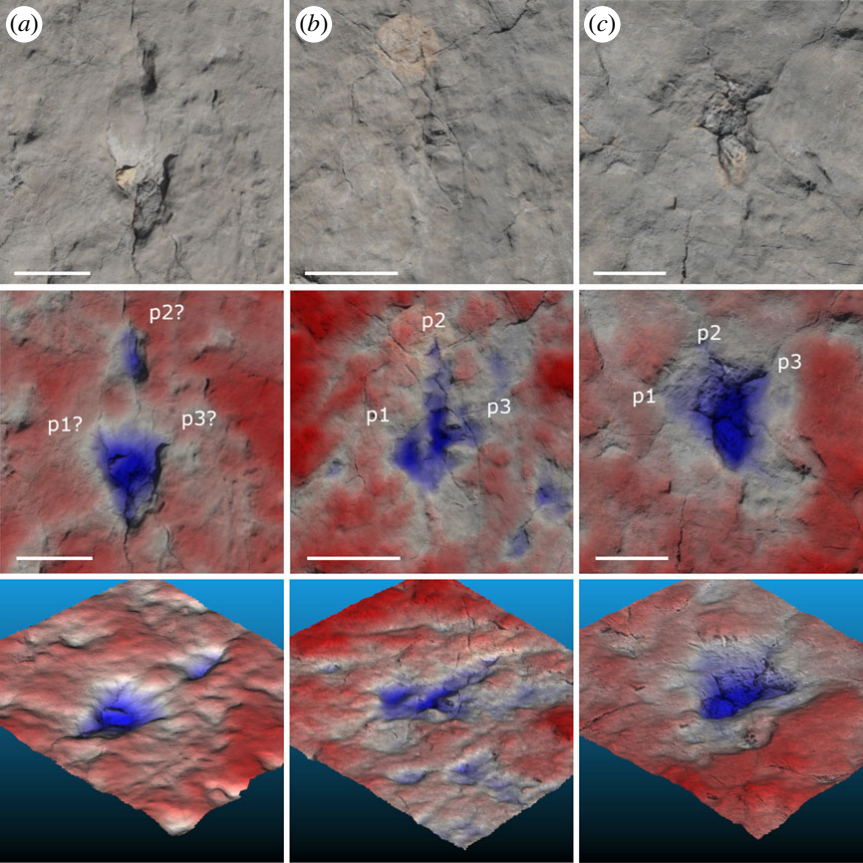
Morphologies in selected tracks from the R2 surface, displaying the true colour photogrammetric reconstruction, false colour relief map and oblique view of the same. Figure 4 for overall track positions on the R2 surface. Inferred pedal positions are labelled with ‘p’ in each track, including ‘?’ if highly uncertain. (*a*) Track 11 in T2. (*b*) Track 10 in T2. (*c*) Track 3 in T4. All scale bars are 10 cm.

Track morphology differs between the R1 and R2 surfaces. The R1 surface bears a single set of tracks (figure 3). These are larger, deeper, and more strongly ovate than those present on the R2 surface (figure 4), with lunate displacement rims around the same region of each track margin. In the most well-preserved tracks, these rims display an incision aligned with the long axis of the track and narrowing away from the track centre (e.g. tracks 6 and 8; figure 3*a*). The R2 surface bears four sets of tracks, with track morphology varying from broadly ovate to a series of small lobes which overlap at a single central depression, showing the placement of digits. In the better-preserved examples on the R2 surface, these digits clearly indicate a tridactyl track maker (figure 5).

## 5. Discussion

### 5.1. Palaeoenvironmental setting

We confidently reject the hypothesis that the traces are abiotic sedimentary structures and instead interpret them as dinosaur trackways based on their size, age and comparable morphology to other dinosaur trackways around the world, in particular the alternating foot placement in each trackway [41]. This necessitates subaerial exposure of their substrate, but the shift from siliciclastic to carbonate deposition in units I to V marks a transgressive event. We interpret units I and II as floodplain deposits, with the red coloration and calcrete nodules supporting subaerial exposure. In units III and IV, the coarsening-up sequence indicates a rapid increase in depositional energy which we ascribe to a transitional, extremely shallow-water environment. Bivalved shells within the conglomerate comparable to those in the overlying limestone support this interpretation, suggesting that unit IV is a pebble beach at the shoreface. The shell beds within unit V limestone indicate that the area was fully marine at several points, but the thinness of the unit, its high clastic content and the presence of a subaerial trackway show that this transgression was short-lived, and that the shoreline remained relatively close. The short-lived, shallow character nature of the transgression and the proximity to the shore suggests that the trackways were therefore made in a calm, tidal environment that permitted preservation in carbonate-rich mud. This is comparable to the depositional setting for Middle Jurassic dinosaur footprint sites found across Central Asia (table 1), and for many dinosaur trackways globally [42]. The trackway surface is a Type 1 site [43], given the absence of any vertebrate body fossils accompanying the footprints. The presence of dinosaurs in the area, however, raises the potential for future discovery of body fossil material. Dinosaur body fossils and even eggs have been discovered in tidal carbonate facies [44,45], but the more strongly terrigenous floodplain deposits underlying Unit V within the Technikum Formation may be more taphonomically suitable facies within which to prospect for bone.

### 5.2. Trackway morphology and biomechanics

The larger R1 surface trackway does not display digit impressions, complicating inference of the track maker identity. While its alternating offset could indicate a bipedal track maker, the width of this offset does not eliminate a narrow-gauge quadruped, and the elongate morphology of some tracks (e.g. track 4; figure 3*a*) could be attributed to quadrupedal overstepping (where the placement of the pes overlaps with the track left in the front by the corresponding manus). The tracks do not display the pronounced circular displacement rims associated with sauropod trackways. Instead, the lunate displacement rims indicate the forward direction of travel (figure 3*a*) and so the cross-cutting incisions aligned with the central axis of the tracks may represent drag marks from a centrally aligned toe or toe claw. This favours a tridactyl biped as the track maker for the R1 tracks.

Some of the R2 tracks show incised lobes likely corresponding to digit impressions, allowing them to be more confidently ascribed to small-bodied tridactyl bipeds based on their size and the gross morphology of the better-preserved prints. None of the trackways are well preserved enough to consistently show homologous digit impressions from track to track, but in a few cases, they are clearly tridactyl with other tracks occasionally showing more elongate profiles where the metatarsals contacted the substrate at the back of the track as the foot sank deeply (e.g. track 1 in R2-T3; figure 5*c*) [46]. These features indicate the direction of travel for each trackway. Following Thulborne [47], we calculate hip height (H) from pedal length (PL) assuming a bipedal, non-avian dinosaurian affinity for the track makers (but see below) using the equations for small-bodied (*H* = 3.06 × PL^1.14^) and large-bodied bipedal dinosaurs (*H* = 8.60 × PL^0.85^), taking a pedal length < 35 cm as the threshold for small body size. Body length (*L*) is then calculated from hip height for small-bodied (*L* = 4H) and large bodied bipedal dinosaurs (*L* = 2H + 3.5). Three different body size classes are represented at the R2 surface, with two of the four trackways differing only fractionally (table 3), suggesting that the R2 trackways were made by at least three individuals rather than a single trackmaker repeatedly traversing the surface. In addition to these individuals, the substantial difference in track size of the R1 traces indicates the presence of a fourth bipedal tridactyl individual.

Following Alexander [48], we calculate gait for each trackway as stride length/hip height. In all cases, gait indicates a walking pace (gait < 2; table 3). For the R1 trackway (assuming the apparent bipedalism is not confounded by potential overstepping) and three of the four trackways on the R2 surface, there is clear alternation of left and right prints along their total lengths. For one trackway on the R2 surface, however, there are instances where the regular walking gait is interrupted. This corresponds to two consecutive depressions which are broader than single footprints (tracks 4 and 5 in R2-T2; figrue 4*a*), then a third pair of pedal impressions which are distinct, but almost touching (tracks 6 and 7 in R2-T2; figure 4*a*). We interpret these irregularities as instances where the individual briefly paused and matched its foot positions, producing the broader depressions in the substrate, before resuming its walking gait.

### 5.3. Track maker identity

The lack of clear morphological features in the R1 traces makes it difficult to distinguish whether the R1 and R2 individuals are of different ontogenetic stages within the same species or different species entirely, and the extent to which morphological differences between the tracks reflect original pedal morphology versus taphonomic distortion is not immediately obvious. Differences might be attributed to exposure of surface impressions and transmitted under-tracks in the same horizon. The presence of relatively fine detail in both trackways (claw drags in the R1 tracks, digits in the R2 tracks) in conjunction with the depth of the R1 tracks, however, suggests that both sets of tracks are original surface structures. Larger bodied, heavier trackmakers will produce deeper impressions that are more prone to slumping, particularly when made wet sediment, as the weight of the surrounding substrate overcomes its competency [40]. This is congruent with our inference of a large body size and so a high body mass in the R1 tracks and could explain the lack of clear digit impressions compared to the R2 tracks but is difficult to justify given the presence of fine morphological detail. Instead, we posit that despite their poor preservation overall, the R1 and R2 trackways represent different taxa.

The lack of digit impressions in the presumably tridactyl R1 tracks complicates determination of a theropod or ornithopod affinity. While the R2 trackways display more detail, they are still poorly preserved and there are too few tracks with clear digits to statistically infer a theropod or ornithopod affinity from interdigitation angle [49]. Despite the lack of precise morphological detail, however, the diverging orientations of the tracks and their near-midline placement in both the R1 and R2 trackways suggests that a theropod affinity is more likely overall, with the size of the former confidently marking them out as non-avian. The majority of Mesozoic bipedal tridactyl trackways are assigned to ornithopods or non-avian theropods, but birds also create trackways of this form. Generally, Mesozoic avian tracks are noticeably smaller than dinosaur tracks (less than 10 cm pedal length), reflecting the smaller body sizes associated with powered flight, but a few Mesozoic bird tracks overlap in size with the R2 trackways [50]. A large tridactyl track from the Cretaceous of central China was attributed to the avian ichnogenus *Magnoavipes* [51], although this may alternatively have been made by an ornithomimid dinosaur [50]. More pertinently, at least one species of bird with a body size potentially approaching that of the modern-day ostrich has been described from the Upper Cretaceous of Kazakhstan [52], with ostriches displaying stride lengths that readily overlap with the stride lengths in the R2 trackways [53]. The biogeographic proximity and potential body size of such a species raise the possibility that the R2 trackways could have been made by birds. Nonetheless, the presumably greater abundance and diversity of cursorial non-avian theropods in Cretaceous assemblages would favour them as the more likely track makers.

### 5.4. Trackway discovery and geoconservation

Earthquake-triggered landslides frequently occur around Mailuu Suu, exacerbated by water lubricating the contact between an underlying, impermeable lithology and an overlying, slippage-prone lithology, and exemplified by the relationship between the durable limestones and more porous siliciclastic units in this area [31]. Slippage of the lowermost layers of the Isolith ‘formation’ overlying the trackway surface were responsible for its exposure. This unit is the second most susceptible formation to landslides in the region [31], so future landslides in this unit may conceivably expose larger portions of the limestone surface, increasing the potential for further discoveries of trackways. While landslides may expose trackways in the future, the unstable nature of the slopes in the Mailuu Suu area will also increase the rate of track loss. Blocks of the track surface have already sheared away, so the photogrammetric data generated by this study will be essential to their long-term conservation.

Minimally, our digital models provide a stable record of the tracks which can also be used to accurately assess the extent of future damage to the surface using the same imaging method [54]. This lightweight, accessible resource can also be used to disseminate palaeontological heritage in Kyrgyzstan. More broadly, photogrammetry and similar low-cost imaging methods have the potential to provide stable, minimal maintenance records of key palaeontological sites and specimens. This will benefit geoconservation efforts in the country given the current paucity of native Kyrgyz palaeontologists or national legislation pertaining to fossils, despite the wealth of fossil data the country has to offer.

## 6. Conclusion

We present the first record of dinosaur trackways in Kyrgyzstan, exposed within the Upper Cretaceous Nichkesai Formation near Mailuu Suu city by a landslide. Owing to the vulnerability of the trackways to damage and loss in future Earth movements, we digitally conserve and analyse them using photogrammetry. The five trackways, split between two areas of the trace-bearing surface record the presence of at least four bipedal, tridactyl individuals which traversed the muddy surface of carbonate shoreline or tidal flat, a common setting for many Mesozoic-aged dinosaur trackways, at a walking pace. The relatively poor preservation of the trackways precludes precise taxonomic identification of their makers, but at least two different taxa were likely present based on the markedly larger pes size and gross morphology of one of the trackways. This trackway was presumably made by a large-bodied ornithopod or non-avian theropod dinosaur. The other four trackways are smaller and were likely made by smaller-bodied ornithopods or theropods. The presence of potentially gigantic avians in Late Cretaceous Central Asia raises the possibility that, for the latter affiliation, the track makers could have been either avian or non-avian. We favour a non-avian track maker, however, based on their presumed frequency over avian dinosaurs in Cretaceous assemblages. Finally, the high frequency of landslides in the Mailuu Suu area and particularly within the unit overlying the trackway surface, have potential to uncover new trackways in the future and provider the finer anatomical detail needed to resolve the affinities of the track makers more precisely. The presence of dinosaur tracks also raises the potential for corresponding future discoveries of body fossil material.

## Data Availability

All data generated by this study are archived on Zenodo [39].
